# Systemic thrombo-embolic events in a middle-aged male with Loeffler endocarditis without peripheral eosinophilia—a case report

**DOI:** 10.1186/s12872-022-02911-3

**Published:** 2022-12-12

**Authors:** Mintje Bohné, Sebastian Bohnen, Hans-Christian Voigt, Hendrik van der Schalk, Da-Un Chung, Stephan Willems, Karin Klingel, Dietmar Kivelitz, Edda Bahlmann

**Affiliations:** 1 Department of Cardiology, Asklepios Clinic St. Georg, Lohmühlenstraße, 20099 Hamburg, Germany; 2 Department of Vascular and Endovascular Surgery, Asklepios Clinic St. Georg, Hamburg, Germany; 3 Cardiopathology, Institute for Pathology, University Hospital Tübingen, Tübingen, Germany; 4 Department of Radiology, Asklepios Clinic St. Georg, Hamburg, Germany

**Keywords:** Loeffler Endocarditis, Thromboembolic events, Case report

## Abstract

**Background:**

Loeffler-endocarditis (LE) is considered a chronic restrictive cardiomyopathy and manifestation of eosinophilic myocarditis characterized by eosinophilic infiltration. LE is a rare underdiagnosed disease and associated with high morbidity and mortality.

**Case presentation:**

We report a case of a 46-year-old man suffering from LE associated with thromboembolic events without peripheral eosinophilia. The patient presented with typical clinical signs of acute onset of limb ischaemia, predominantly on the right limb, indicating immediate iliacal thrombectomy and due to a severe compartment syndrome additional fasciotomy. Total occlusion also of left popliteal artery suggesting an impaired chronic and aggravated impaired perfusion indicated also urgent left sided revascularization. Subsequent echocardiography revealed severe left ventricular dysfunction with a striking amount of spontaneous echo-contrast, noticeable in the left ventricular cavity. Furthermore the initial CT scan demonstrated asymptomatic left kidney- and brain infarctions. Diagnostic workup including endomyocardial biopsy (EMB) of the left ventricle, uncovered an underlying LE without peripheral eosinophilia.

**Conclusions:**

This case demonstrates and highlights the findings, treatment and outcome of a patient with LE and associated thrombo-embolic events without peripheral eosinophilia and emphazises the importance of awareness for LE in patients presenting with an acute cardiac decompensation and thrombo-embolic events. EMB should be performed early in unstable patients unsuitable for cardiovascular magnetic resonance imaging.

**Supplementary Information:**

The online version contains supplementary material available at 10.1186/s12872-022-02911-3.

## Background

Endocarditis parietalis fibroplastica or Loeffler-endocarditis (LE) is a rare, underdiagnosed idiopathic systemic disease, first described in 1936 [1]. The also called Loeffler cardiomyopathy or endomyocarditis is considered as chronic restrictive cardiomyopathy and is a manifestation of eosinophilic myocarditis, characterized by eosinophilic infiltration, and often accompanied by peripheral eosinophilia [2–5]. In patients with hypereosinophilic syndrome, sustained overproduction of eosinophils with cardiac manifestation is described in about 50% [6]. Typically, hypereosinophilic syndrome is defined by the combination of unexplained prolonged blood eosinophilia of greater than 1.500/µL in the absence of parasitic infections and allergic diseases and/or evidence of organ involvement [7–9], affecting mostly male subjects between 20 and 50 years of age [7, 8, 10–13]. Thrombo-embolic events relating to the high incidence of left ventricular (LV) thrombi in eosinophilic myocarditis, described in 28.3%, are serious complications [14]. Suspecting the diagnosis of LE in a case with normal serum eosinophil count, reported in 24% of cases with eosinophilic myocarditis is challenging [13, 14].

## Case presentation

### Timeline

**Table:** 

Time	Events
Day 1	Presentation with typical clinical signs of acute onset of limb ischemia, predominantly on the right limb Contrast enhanced computed tomography (CT) showed a complete occlusion of the distal right common iliac artery, right external and internal iliac artery and common femoral artery Furthermore, an occlusion of the left profound femoral artery was found and an asymptomatic subacute left sided kidney- and small right-sided posterior cerebral infarction Transesophageal echocardiography revealed severe systolic LV dysfunction and a striking amount of spontaneous echo-contrast Open thrombectomy of the right limb combined with full fasciotomy on the right calf was performed Antithrombotic therapy with continuous unfractionated heparin was startet
Day 2	Development of a compartment syndrome of the right thigh and clinical detereoration of left limb arterial perfusion Relieve of the massive compartment syndrome Revascularization of femoral and popliteal artery and all crural vessels, endarterectomy and patch of popliteal and tibiofibular artery as well as stent-angioplasty of the popliteal artery and angioplasty of the anterior and posterior tibial artery and fasciotomy of the left calf was performed Laboratory results revealed markers suggesting rhabdomyolysis with highly increased creatinine kinase and acute renal failure
Day 2 until discharge	Acute renal failure was treated with continuous hemofiltration following renal replacement with intermittent dialysis
Day 6	Cerebral CT scan was noticeable for an asymptomatic small right-sided posterior cerebral infarction Coronary angiography showed no underlying coronary heart disease and LV endomyocardial biopsy was performed
Day 11	The diagnosis of an underlying Loeffler endocarditis (LE) was established by results of LV endomyocardial biopsy
Day 12	Immunosuppressive therapy with prednisolone was initiated and continued for 14 days, followed by a dose tapering regimen of 10 mg every four weeks in combination with azathioprine 300 mg/day
Day 14	Secondary wound closure of fasciotomy sites could be achieved and both limbs salvaged
Day 23	A Demers catheter was implanted
Day 40	Diagnosis of SARS CoV2 infection
Day 43	The patient was equipted with a LifeVest® as wearable cardioverter defibrillator to protect patients at risk of sudden cardiac death
Week 9	Last follow-up echocardiography revealed an unchanged LV dysfunction with an estimated ejection fraction of 34% and decreasing spontaneous echo contrast. Heart failure medication in combination with anticoagulation and continued immunosuppressive therapy was continued
Week 12	The patient was discharged from hospital

**Fig. 1:**
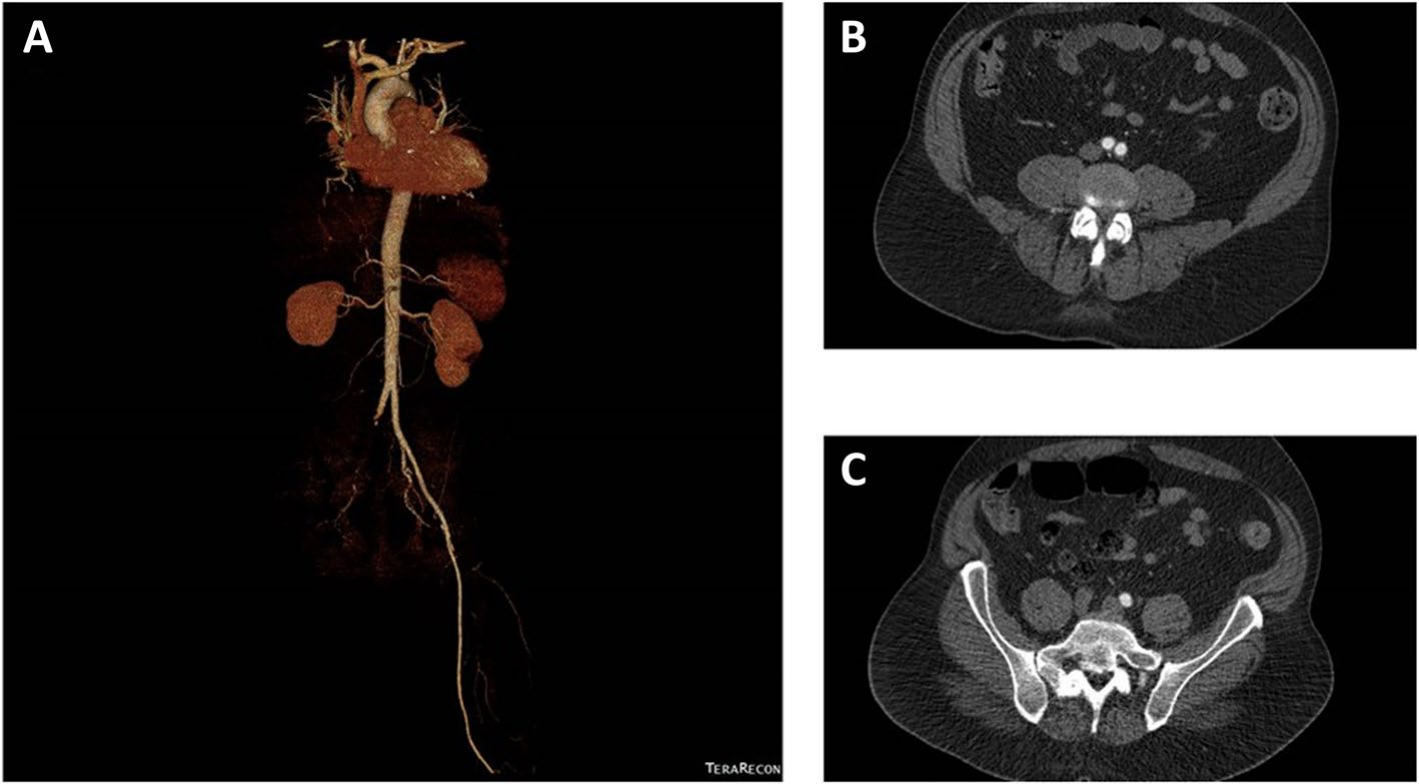
Three dimensional Volume Rendering Technique (VRT) of the computed tomography angiogram demonstrating **A** right-sided pelvic occlusion, and **B** axial computed tomography scan demonstrating both pelvic arteries and **C** only the left-sided arteria iliaca communis and right-sided occlusion of the arteria iliaca communis

**Fig. 2:**
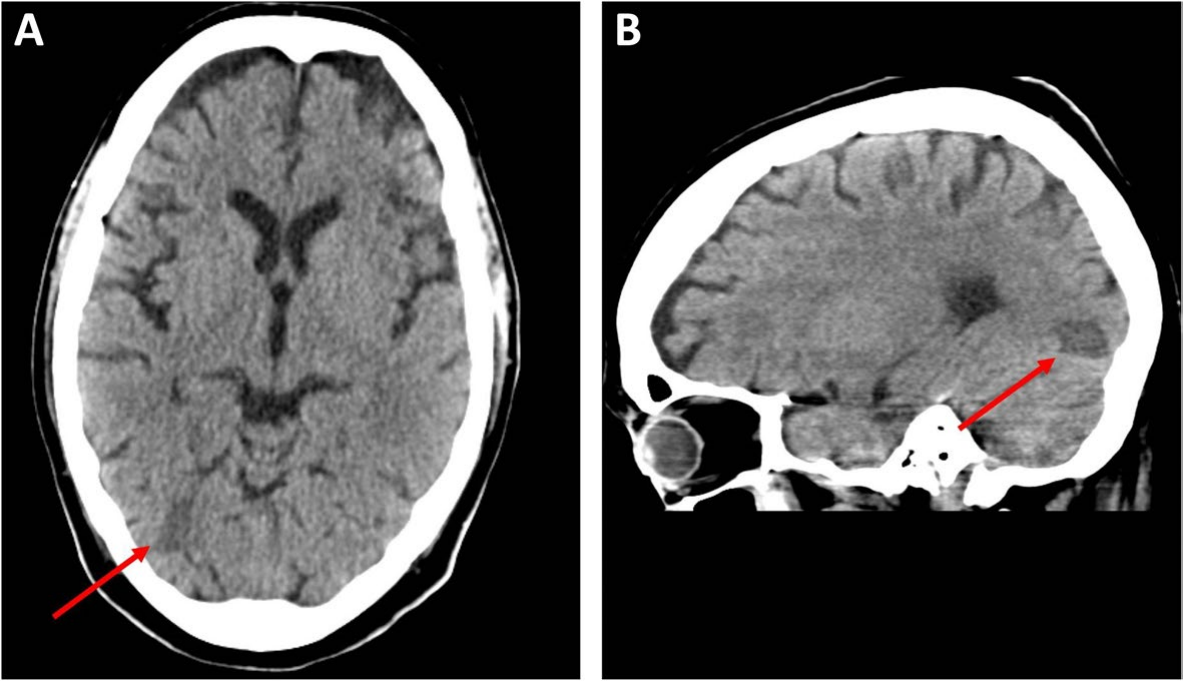
Computed tomography scan of the brain showing a small right sided posterior infarct, **A** transversal and **B** sagittal (red arrows)

**Fig. 3:**
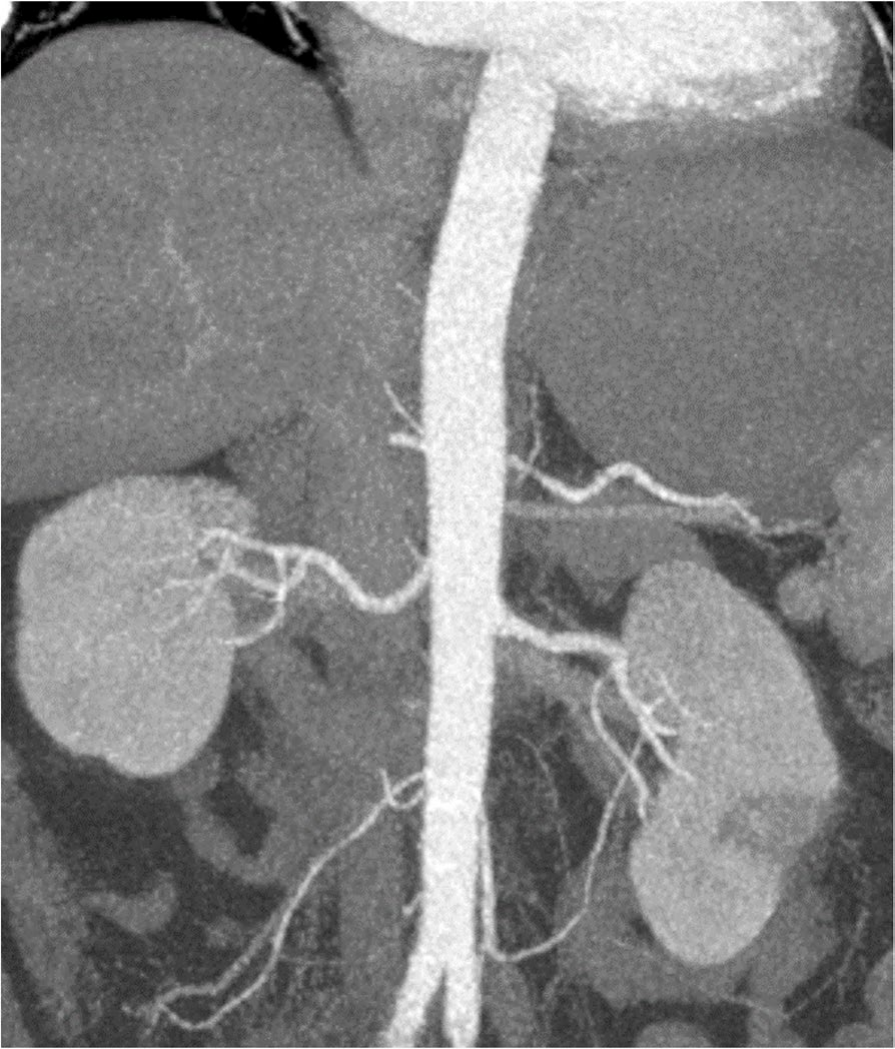
Computed tomography angiography showing a left sided kidney infarct

**Fig. 4:**
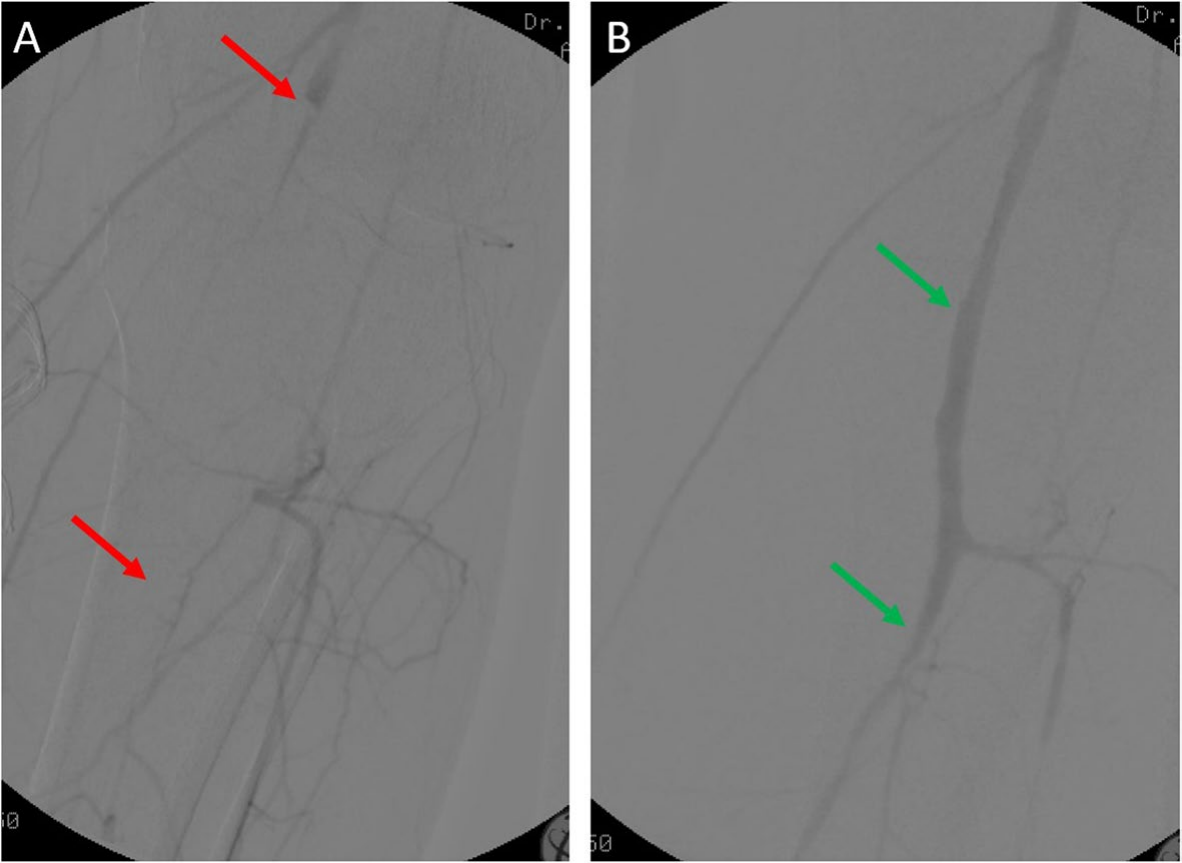
Intraoperative angiography prior to revascularization showing complete occlusion of left popliteal artery, peroneal and posterior tibial artery (red arrows) (**A**). Angiography after complex hybrid revascularization including open thrombectomy, angioplasty, endarterectomy and patch popliteoperoneal showed reestablished popliteocrural perfusion (green arrows) (**B**)

**Fig. 5:**
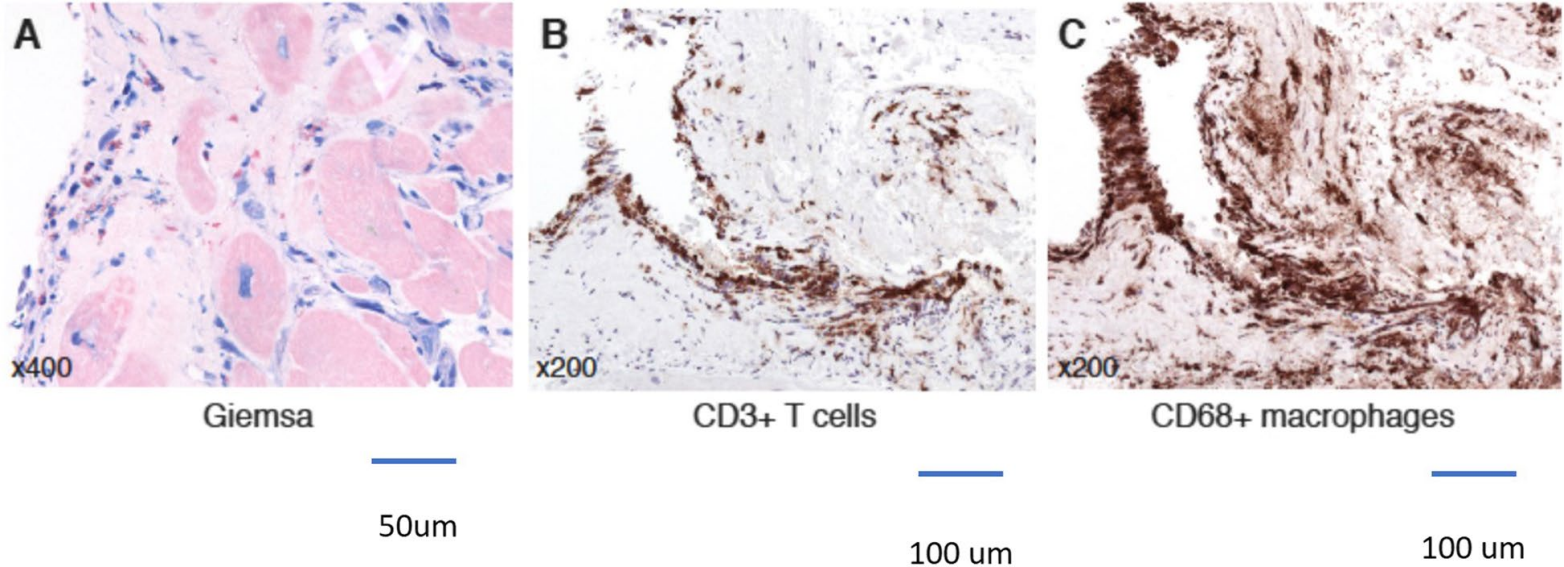
Histological/immunohistological findings in the endomyocardial biopsy showing eosinophilic granulocytes (Giemsa stain × 400) (**A**), CD3 + T-cells (× 200) (**B**) and CD68 + macrophages (× 200) (**C**). Resolution of images: 1,5 MB. Microscope: Axioskop 40 (Zeiss Jena), Camera: ProgRes C10 (Jenoptik Jena), acquisition software: Imagic IMS Client (Imagic Bildverarbeitung AG, Glattbrugg, Schweiz).

A 46 years old, previously healthy man presented with typical clinical signs of acute onset of limb ischemia (pain, paleness, pallor, pulselessness, paralysis and paresthesia), predominantly on the right limb. An emergency contrast enhanced computed tomography (CT) scan of the complete aorta to thighs was performed, based on the initial suspicion of an aortic dissection. An aortic dissection however was ruled out. Main findings were a complete occlusion of the distal right common-, the right external- and internal iliac artery and common femoral artery. Furthermore, an occlusion of the left profound femoral artery and mild arteriosclerosis was found, the occluded vessel segments however were characterized by hypodense thrombotic material (Fig. 1). The CT scan, in addition, was noticeable for an asymptomatic subacute left sided kidney- and small right-sided posterior cerebral infarction (Figs. 2 and 3). Patients` history was positive for moderate nicotine consumption. The patient denied dyspnea, fatigue, cough, fever, orthopnea/paroxysmal nocturnal dyspnea, stroke related symptoms, chest pain, or lower extremity edema. The patient was on no long-term medication. On admission, he presented with an increased respiratory rate of 20/min, hypertensive blood pressure of 161/105 mmHg and a normal heart rate of 74/min and an oxygen saturation of 97%. Electrocardiogram showed sinus rhythm. Transthoracic echocardiography demonstrated moderate biventricular enlargement and severe systolic LV dysfunction with an estimated ejection fraction of 35% without signs of wall thickening, valvulopathy or pericardial effusion. Further clarification for the source of embolism and due to a poor transthoracic acoustic window, additional transesophageal echocardiography was justified and revealed a striking amount of spontaneous echo-contrast in the LV cavity (Video 1). There were no signs of LV wall thickening, valvulopathy or pericardial effusion. Initial laboratory testing was noticeable for a slightly elevated lactate of 3.2 mmol/l, rapidly increasing to a maximum of 6.3 mmol/l and normalizing post intervention and a leukocytosis of 11/nl, increasing to a maximum of 24/nl 2 days post admission and likewise normalizing thereafter. The patient was directly taken to the operation theater and open thrombectomy via femoral access was performed on the right limb, in combination with full fasciotomy on the right calf. On the next day, a compartment syndrome of the right thigh developed as well as clinical detereoration of the left limb arterial perfusion. Therefore, fasciotomy of the right thigh and revascularization of the left limb was indicated and a massive compartment syndrome on the right thigh was relieved. Total occlusion also of left popliteal artery suggesting an impaired chronic and aggravated impaired perfusion indicated also urgent left sided revascularization (Fig. 4). Revascularization of the left limb was complex, due to a mixture of fresh and older sticky and organized harder thrombus, inducing scarring and vessel shrinking in combination with a perivascular inflammatory reaction. Revascularization however was achieved by hybrid surgery in terms of open thrombectomy of femoral and popliteal artery and all crural vessels, endarterectomy and patch of popliteal and tibiofibular artery. Additionally, stent-angioplasty of the popliteal artery and angioplasty of the anterior and posterior tibial artery and fasciotomy of the left calf was performed. Post-surgical laboratory results revealed markers suggesting rhabdomyolysis with a highly increased creatinine kinase of up to 206000U/l and acute renal failure with an elevation of creatinine of 4.7 mg/dl. Further laboratory work-up revealed no peripheral eosinophilia. Perinuclear anti-neutrophil cytoplasmic antibodies (p-ANCA) and cytoplasmic anti-neutrophil cytoplasmic antibodies (c-ANCA) were negative. In addition, laboratory testing was negative for antiphospholipid syndrome and heparin-induced thrombocytopenia and showed normal levels for protein C and S. Further investigation by a coronary angiography on day 6 showed no coronary heart disease. The diagnosis of an underlying LE was established by subsequent LV endomyocardial biopsy (EMB). Histological findings present on day 11 were suggestive for LE, and showed increased amounts of CD3 T-cells, CD68 + macrophages and eosinophilic granulocytes in the endocardium (Fig. 5). Due to an increased body mass index of 41 and patients disability to endure, Cardiac Magnetic Resonance (CMR) imaging had to be terminated prematurely and data could not be obtained. Acute renal failure was treated with continuous hemofiltration, following renal replacement with intermittent dialysis. On day 23, a Demers catheter was implanted. Additionally, a beta-blocker medication was started. Immunosuppressive therapy with prednisolone (1 mg/kg/day) was initiated on day 12 and continued for 14 days, followed by a dose tapering regimen of 10 mg every four weeks in combination with azathioprine (300 mg/day). Because of an increase in inflammatory markers, most likely in the context with a decubital ulcer, azathioprine therapy was temporarily discontinued and prednisolone therapy was reduced. Due to the high risk of systemic embolization, antithrombotic therapy with continuous unfractionated heparin was immediately started on day 1 and switched to Phenprocoumon (vitamin K antagonist). In the further course secondary wound closure of fasciotomy sites could be achieved and both limbs salvaged.

After the patient had gone through a SARS CoV2 infection he was finally discharged from hospital free of symptoms 8 weeks after admission. Last follow-up echocardiography revealed an unchanged LV dysfunction with an estimated ejection fraction of 34%, associated moderate LV hypertrophy and decreasing spontaneous echo contrast. Heart failure medication in combination with anticoagulation and continued immunosuppressive therapy including prednisolone and azathioprine was continued. The patient was equipted with a LifeVest® as wearable cardioverter defibrillator to protect patients at risk of sudden cardiac death.

## Discussion and conclusions

To our knowledge, this is the first report of multiple thrombo-embolic events in a patient with LE without peripheral eosinophilia. It provides new information on the natural history of this rare but clinically relevant disease.

### Pathophysiology

In LE cardiac involvement with migration of eosinophils into the myocardium follows a stepwise pattern from subclinical acute necrosis of the myocardium [15], thrombosis as a result of damage to the endomyocardial surface and fibrosis [8, 16]. Typically, this leads to a restrictive or as in our patient dilated cardiomyopathy [17, 18].

### Clinical presentation and diagnostic

Noninvasive multimodality imaging, including transthoracic- and transesophageal echocardiography as the mainstay of diagnostic imaging and surveillance for LE could, in our case, not provide classic findings as LV wall thickness, valvulopathy or LV thrombus [6, 10, 18]. Rather, the pattern of a dilated cardiomyopathy with LV enlargement and severely impaired LV function was detected [14]. Impaired tissue and organ function cause high morbidity and mortality [3]. Prognosis in LE therefore is poor due to high mortality from heart failure, sudden cardiac death or thrombo-embolism due to LV thrombus formation [12, 19, 20]. In a prospective study of 25 patients by Parrillo JE et al. [21], dyspnea was the most common symptom (42%), followed by chest pain (27%), heart failure (38%), cough (12%), palpitations (8%) and thrombo-embolic events (4%). Analysis of 26 case reports approved these findings, although thrombo-embolic events, predominantly presented as stroke related symptoms, was specified with a higher prevalence of 15% [11]. Among 33 cases of LE associated with LV thrombus, the incidence of an embolic stroke increased to 36.4% with a high mortality rate of 27.3% [8]. LV thrombus formation was found in 24% and right ventricular thrombus in 20%, documented in 55 patients with hypereosinophilic syndromes by a study from the Mayo clinic [22]. The evidence of a striking amount of spontaneous echo-contrast in transesophageal echocardiography resulting from low-velocity blood flow might, as discussed in the literature, be the underlying cause of thrombo-embolic events in our case, as LV thrombus formation was not detectable by echocardiography [18, 23]. It is also believed that eosinophils may contribute to thrombus formation by binding to thrombomodulin and impairing the inherent anticoagulant properties of the endothelial membrane [24]. CMR imaging is a powerful noninvasive modality in providing diagnostic and follow-up information in these patients, in particular if suspicious for the diagnosis of LE with thrombus formation, however, not possible in our case [12, 18]. EMB remains the gold standard, confirming the diagnosis also in our case, although containing risks, such as sampling errors or iatrogenic embolism [18, 25].

### Medical therapy

A beneficial effect of immunosuppressive therapy with prednisone and azathioprine with significant improvement of LV function and decrease of LV dimensions was first shown in a randomized placebo-controlled study by Frustaci et al. in 2009 [26]. In the study by Brambatti et al., however, 78% of patients with eosinophilic myocarditis received corticosteroids and a minority had an additional immunosuppressant, however, uncertainty remained to what degree immunosuppression affected outcome [14]. The treatment of LE in particular, is mainly based on case reports and small case series and guidelines or consensus statements for the treatment of eosinophilic myocarditis/LE are still missing [27]. Aiming the optimal therapeutic strategy for our patient, immunosuppressive therapy was started after confirming the diagnosis by EMB. Considering LE patients with an intracardiac thrombus formation in a review including 32 studies, steroids were administered in 81.8% of patients, achieving a rapid decrease in the eosinophil count [12]. As no peripheral eosinophilia was present in our case, this therapeutic effect was not measurable.

The goal of recommended medical therapy in LE, including heart failure management and anticoagulation if presence of an intracardiac thrombus, is a decrease of eosinophil-mediated end-organ damage and prevent adverse thrombotic events [11, 12, 16, 28]. In our patient, presenting with clinical signs of acute heart failure and severely reduced LV function, medical heart failure therapy was started immediately, but was limited to a ß-blocker due to the need of renal replacement. In addition, our patient was put on continuous therapeutic anticoagulation promptly after presenting with systemic thrombo-embolic events and a striking amount of spontaneous echo-contrast in echocardiographic imaging. In analogue with the high amount of 69.7% of patients treated with anticoagulant therapy with a detectable decrease in thrombus mass [12], the amount of detectable spontaneous echo contrast decreased in echocardiographic follow-up study in our patient, although LV systolic function remained severely impaired. The role of anticoagulation, however, in patients with spontaneous echo-contrast and to prevent endocavitary thrombus formation is unclear and might be of high interest in patients with LE.

### Risk assessment

Decision for premature implantable cardioverter defibrillator system remain challenging in patients with impaired LV function (ejection fraction ≤ 35%), but should be avoided in patients with inflammatory cardiomyopathy as LV function may improve significantly with guideline-based heart failure therapy [4]. Close surveillance is necessary in patients like ours.

## Conclusion

In conclusion, LE without evidence of peripheral eosinophilia and absence of intracardiac thrombus formation, as demonstrated in this unique patient report, can be associated with life-threatening systemic thrombo-embolic events. Our case highlights the importance of considering LE as an important differential diagnosis in the setting of an acute cardiac syndrome in combination with thrombo-embolic events and helps to identify characteristic features found in patients with LE.

For strategic recommendation based on the experience in our particular case, we suggest in a patient suspicious for LE the following take-home messages:

- early multimodality imaging including transesophageal echocardiography and if possible CMR imaging to explore not only the presence of an LV thrombus but also possible spontaneous echo-contrast to consider immediate anticoagulation aiming to prevent thrombo-embolic events
- EMB should be performed early in patients with severely impaired LV dysfunction of unknown etiology
- Immunosuppressive therapy should be initiated promptly in a patient with proven LE

## Supplementary Information

**Additional file 1: Video 1.** Transesophageal echocardiography showing severe systolic LV dysfunction and a striking amount of spontaneous echo contrast but no evidence of LV thrombus in a 3-chamber view (video clip).

## Data Availability

Data and material is presented within the manuscript.

## Abbreviations

EMB: Endomyocardial biopsy
LE: Loeffler-endocarditis
LV: Left ventricle
CMR: Cardiac Magnetic Resonance
CT: Computed tomography
